# Effects of supplementary butyrate on butanol production and the metabolic switch in *Clostridium beijerinckii* NCIMB 8052: genome-wide transcriptional analysis with RNA-Seq

**DOI:** 10.1186/1754-6834-6-138

**Published:** 2013-09-27

**Authors:** Yi Wang, Xiangzhen Li, Hans P Blaschek

**Affiliations:** 1 Department of Food Science and Human Nutrition, University of Illinois at Urbana-Champaign, Urbana, IL 61801, USA; 2 Institute for Genomic Biology, University of Illinois at Urbana-Champaign, Urbana, IL 61801, USA; 3 Key Laboratory of Environmental and Applied Microbiology, Chengdu Institute of Biology, Chinese Academy of Sciences, Chengdu 610041, China; 4 Center for Advanced Bioenergy Research (CABER), University of Illinois at Urbana-Champaign, Urbana, IL 61801, USA

**Keywords:** Butyrate, Butanol, ABE fermentation, *Clostridium beijerinckii*, Metabolic switch, Solventogenesis, Transcriptional analysis, RNA-Seq

## Abstract

**Background:**

Butanol (n-butanol) has high values as a promising fuel source and chemical feedstock. Biobutanol is usually produced by the solventogenic clostridia through a typical biphasic (acidogenesis and solventogenesis phases) acetone-butanol-ethanol (ABE) fermentation process. It is well known that the acids produced in the acidogenic phase are significant and play important roles in the switch to solventogenesis. However, the mechanism that triggers the metabolic switch is still not clear.

**Results:**

Sodium butyrate (40 mM) was supplemented into the medium for the ABE fermentation with *Clostridium beijerinckii* NCIMB 8052. With butyrate addition (reactor R1), solvent production was triggered early in the mid-exponential phase and completed quickly in < 50 h, while in the control (reactor R2), solventogenesis was initiated during the late exponential phase and took > 90 h to complete. Butyrate supplementation led to 31% improvement in final butanol titer, 58% improvement in sugar-based yield, and 133% improvement in butanol productivity, respectively. The butanol/acetone ratio was 2.4 versus 1.8 in the control, indicating a metabolic shift towards butanol production due to butyrate addition. Genome-wide transcriptional dynamics was investigated with RNA-Seq analysis. In reactor R1, gene expression related to solventogenesis was induced about 10 hours earlier when compared to that in reactor R2. Although the early sporulation genes were induced after the onset of solventogenesis in reactor R1 (mid-exponential phase), the sporulation events were delayed and uncoupled from the solventogenesis. In contrast, in reactor R2, sporulation genes were induced at the onset of solventogenesis, and highly expressed through the solventogenesis phase. The motility genes were generally down-regulated to lower levels prior to stationary phase in both reactors. However, in reactor R2 this took much longer and gene expression was maintained at comparatively higher levels after entering stationary phase.

**Conclusions:**

Supplemented butyrate provided feedback inhibition to butyrate formation and may be re-assimilated through the reversed butyrate formation pathway, thus resulting in an elevated level of intracellular butyryl phosphate, which may act as a phosphate donor to Spo0A and then trigger solventogenesis and sporulation events. High-resolution genome-wide transcriptional analysis with RNA-Seq revealed detailed insights into the biochemical effects of butyrate on solventogenesis related-events at the gene regulation level.

## Background

Biobutanol produced through fermentation employing the solventogenic clostridia has recently attracted a considerable amount of attention due to its value as a promising biofuel and chemical feedstock [1]. *Clostridium beijerinckii* is an important solvent-producing microbe characterized by a typical biphasic acetone-butanol-ethanol (ABE) fermentation. In a typical batch fermentation, acetic acid and butyric acid are produced during the active exponential growth phase. As growth slows down, acids are re-assimilated by the culture and acetone, butanol and a small amount of ethanol are produced [2]. The switch from acidogenesis to solventogenesis is usually associated with induction of solvent formation gene transcription and decreased cell growth and motility. However, the mechanism that triggers the metabolic switch is still elusive.

The level of intracellular undissociated butyric acid (UBA) has long been suggested as a controlling factor in the shift from acid production to solvent production in the metabolic pathway [2]. Terracciano and Kashket determined that solvent production began in *C. acetobutylicum* when the UBA level reached 13–18 mM [3]. Husemann and Papoutsakis suggested that 6–13 mM of UBA was required for the initiation of solventogenesis when the external pH was between 3.7 and 5.0 [4]. Recently, Lee et al. reported that the addition of butyrate into the culture medium for ABE fermentation with *C. beijerinckii* 8052 enhanced solvent production [5]. But in their study, detailed fermentation dynamics were not included, and no transcriptional analysis or enzyme activity assays were performed, thus making it impossible to draw conclusions regarding the effect of butyrate on the metabolic pathway switch from the level of gene transcription.

Transcriptional profiling of the fermentation culture in the presence of external butyrate supplementation may provide biological evidence for the switch from acidogenesis to solventogenesis. As we previously demonstrated, genome-wide gene transcription can be quantified and profiled to an unprecedented resolution and depth using RNA-seq technology [6-9], with various advantages over traditional array-based transcriptional analysis methods [10,11]. Therefore, the objective of this study was to examine the effect of supplemented butyrate in the growth medium on solvent production and the metabolic pathway switch in *C. beijerinckii*. Further insights are expected at the level of gene regulation concerning the biochemical effects of butyrate supplementation on solventogenesis-related events.

## Results

### Growth kinetics and ABE fermentation

**Figure 1:**
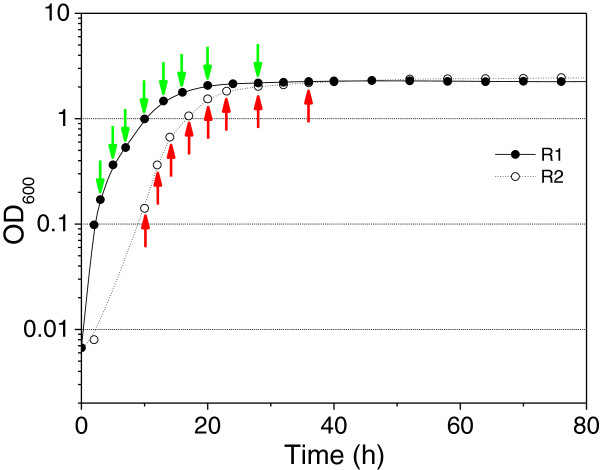
**Cell growth kinetics of *C. beijerinckii *8052 in batch fermentation with 40 mM sodium butyrate addition in R1 and no sodium butyrate addition in R2.** Sampling points for RNA-Seq indicated by arrows.

**Figure 2:**
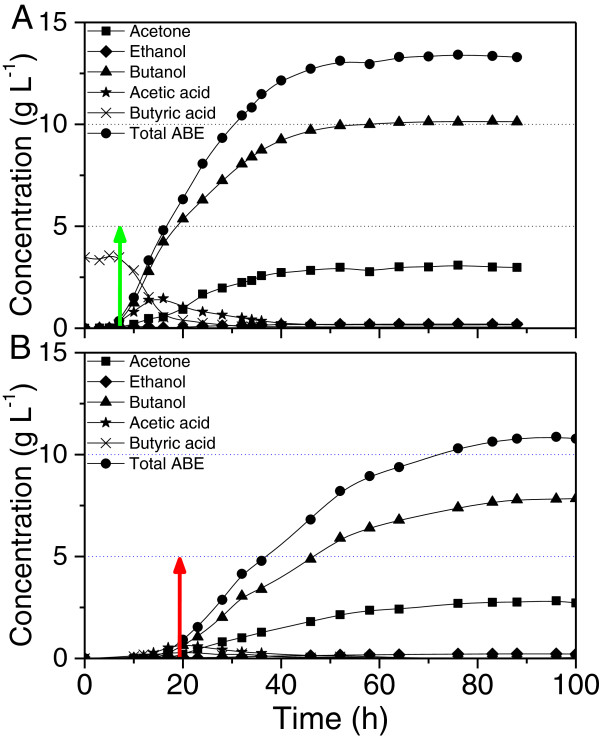
**Solvent and acid production over time with the onset of solvent production indicated by arrows. (A)** Reactor R1: 40 mM sodium butyrate was added to the growth medium. **(B)** Reactor R2: no sodium butyrate was added to the growth medium.

The cell growth response of *C. beijerinckii* in the presence and absence of supplemented 40 mM sodium butyrate is illustrated in Figure 1. The cell culture in the fermentation containing supplemented butyrate (R1) grew very rapidly to the exponential phase, while the control unsupplemented culture (R2) exhibited an obvious lag phase. Both cultures grew to a similar final maximum cell density, although the one in R2 took around 10 hours longer. As shown in Figure 2, in the case of R1, the production of solvents was detected at 7–10 h after the start of fermentation which corresponds to mid exponential growth phase (A_600_ = 0.5). In the case of R2, the fermentation shifted from acidogenesis to solventogenesis at approximately 17–20 h which corresponds to the late exponential growth phase (A_600_ = 1.1) (Figures 1 &2). In R1, the supplemented butyrate was assimilated at a rate corresponding to the solvent accumulation, while the level of acetic acid continued to increase even after the initiation of solvent production for about 6 hours before it reached the maximum level. Solvent production achieved maximum soon after the cell culture reached stationary phase at around 46 h. The unsupplemented fermentation in R2 experienced a switch from acidogenesis to solventogenesis at around 17–20 h. Acetic and butyric acids were the main products produced during the exponential growth phase, although the levels were very low; while acetone and butanol continued to increase throughout the stationary phase beyond 80 h during the fermentation.

With butyrate supplemented into the medium for ABE fermentation with *C. beijerinckii* 8052, solvent production was initiated 10 h earlier and the fermentation completed more rapidly than the control. Butyrate supplementation also led to a higher final butanol titer (10.2 vs 7.8 g L^-1^), yield (0.41 vs 0.26 g g^-1^) and productivity (0.21 vs 0.09 g L^-1^ h^-1^). In addition, the butanol/acetone ratio increased to 2.4 (molar ratio) compared to 1.8 in the control, indicating a dramatic shift in the biochemical pathway towards butanol production.

### RNA-Seq data analysis and overall gene transcription dynamics

RNA samples were taken at eight time points covering different fermentation stages from each reactor, and were sent for sequencing using the Illumina HiSeq 2000 system (Illumina Inc., San Diego, CA). The 100-nt sequence reads were mapped to the *C. beijerinckii* 8052 genome. Only those reads that mapped unambiguously to the genome were used for further analyses (Table 1). Based on the sequence data, 5008 out of 5020 (99.8%) protein-coding genes had detectable expression in all 16 samples. A biological replicate was included in this study to further confirm the effectiveness of expression measurement with RNA-Seq approach. A biological replicate sample was taken at 14 h from a batch fermentation carried out under the identical conditions as for R2. As illustrated in Additional file 1: Figure S1 in the supplemental material, a high degree of correlation (R = 0.969) was observed for the log_2_-transformed RPKM values between sample R23 (taken at 14 h from R2) and the biological replicate sample R23-2 (Table 1). These results further demonstrated that RNA-Seq is an effective approach for quantification of gene expression.

**Table 1:** Summary of RNA-Seq sequencing and data analysis results

**Samples from R1**	**R11**	**R12**	**R13**	**R14**	**R15**	**R16**	**R17**	**R18**	**---**	**Total**
Time collected (h)	3	5	7	10	13	16	20	28	---	
Total number of reads	32934519	29367899	15075431	30451579	37230096	26184184	36117022	32444284	---	241287505
No. of reads mapped	31098649	27046332	14016757	29094448	34907591	24836210	34255629	30754172	---	227240126
No. of reads unambiguously mapped	29844200	26282300	13592284	28326600	34233900	24012800	33747700	30065800	---	221417584
No. of genes with detectable expression^1^	5070	5056	5032	5075	5084	5062	5087	5085	---	5088
Range in expression levels (RPKM)	7.4 × 10^-3^	1.1 × 10^-2^	5.7 × 10^-3^	2.3 × 10^-2^	1.6 × 10^-3^	1.5 × 10^-3^	2.3 × 10^-2^	2.3 × 10^-2^	---	1.5 × 10^-3^
	~ 2.0 × 10^4^	~ 1.8 × 10^5^	~ 8.6 × 10^4^	~ 5.0 × 10^4^	~ 9.2 × 10^4^	~ 1.1 × 10^5^	~ 2.2 × 10^5^	~ 8.8 × 10^4^		~ 2.2 × 10^5^
Samples from R2	R21	R22	R23	R24	R25	R26	R27	R28	R23-2^2^	Total^3^
Time collected (h)	10	12	14	17	20	23	28	36	14	
Total number of reads	25350444	38025575	31352222	33877714	26099516	42485421	38176862	33624379	33916811	268992133
No. of reads mapped	23995017	35773620	29542453	32664767	24890605	40877908	36397654	31652246	32399849	255794270
No. of reads unambiguously mapped	22098900	31363000	27534800	31668100	24198000	39820500	35617800	30760200	31451300	243061300
No. of genes with detectable expression^1^	5059	5063	5058	5077	5084	5088	5088	5084	5081	5088
Range in expression levels (RPKM)	2.3 × 10^-2^	6.2 × 10^-3^	2.4 × 10^-2^	1.6 × 10^-3^	2.8 × 10^-2^	4.7 × 10^-2^	2.6 × 10^-2^	3.6 × 10^-2^	2.9 × 10^-2^	1.6 × 10^-3^
	~ 1.1 × 10^5^	~ 1.0 × 10^5^	~ 1.3 × 10^5^	~ 1.5 × 10^5^	~ 4.6 × 10^4^	~ 6.7 × 10^4^	~ 1.3 × 10^5^	~ 1.2 × 10^5^	~ 1.1 × 10^5^	~ 1.5 × 10^5^

^1^Pseudogenes included.

^2^R23-2 is a biological replicate for sample R23; it was taken at the same fermentation time as for R23, but from a replicate reactor operated under the identical conditions.

^3^R23-2 is excluded.

**Figure 3:**
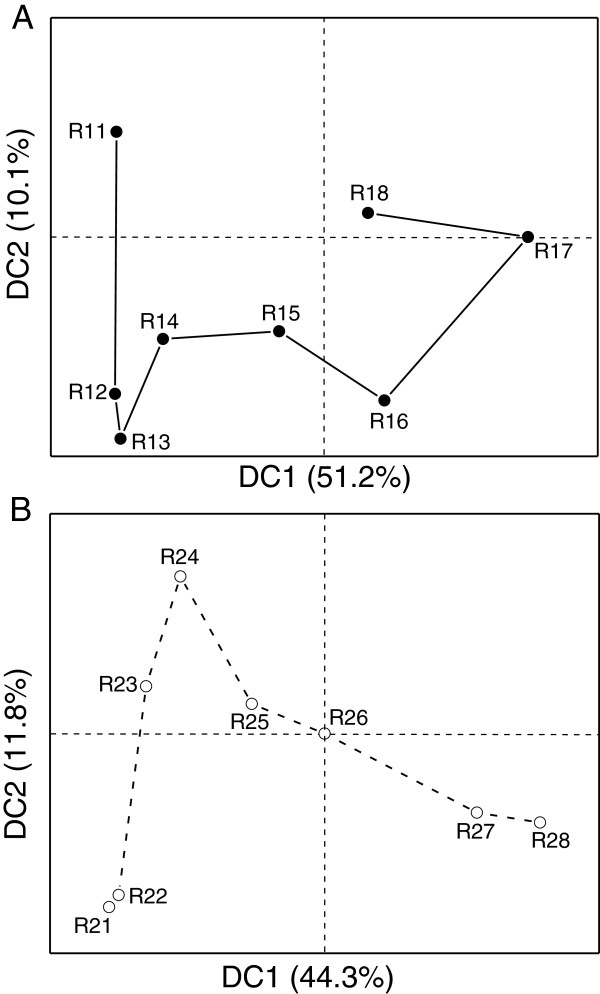
**Detrended Correspondence Analysis (DCA) indicating different overall gene transcriptional dynamics in R1 and R2. (A)** The gene transcriptional dynamic profile in R1 indicated by DCA. **(B)** The gene transcriptional dynamic profile in R2 indicated by DCA.

Detrended Correspondence Analysis (DCA) was conducted on the RNA-seq data as described previously [9], in order to compare the overall difference in dynamic transcription profiles throughout batch fermentation in both reactors. DCA is an ordination technique that uses detrending to remove the ‘arch effect’ usually observed in correspondence analysis [12]. As shown in Figure 3, when depicted using the first two detrended correspondences, the overall gene transcriptional dynamic profiles in R1 and R2 both demonstrated clear, but very different temporal variation trends, which may be indicative of different cell physiology and metabolism under different fermentation conditions.

Sample R13 (taken at 7 h from R1) and sample R24 (taken at 17 h from R2) were taken at the time points when butanol was first detected in R1 and R2, respectively (around 0.2 g L^-1^ in both reactors), and therefore these two time points can be considered as the onset of solventogenesis in R1 and R2, respectively. The expression values of the highly differentially expressed genes from these two samples were plotted against each other in Additional file 2: Figure S2 (and these genes are also listed in Additional file 3: Table S1). Significantly, in R13, the sugar phosphotransferase system (PTS) encoding gene cluster (Cbei_4911-4914, encoding PTS system mannose/fructose/sorbose family IIA-D subunit), and another PTS encoding gene (Cbei_1844, encoding PTS system, fructose subfamily, IIC subunit) were much higher expressed than those in R24. One 1-phosphofructokinase (pfk) encoding gene (Cbei_1843) was also differentially higher expressed in R13. These results correspond to the more active sugar metabolism and glycolysis in R1 at this time point. Two CoA biosynthesis related genes (Cbei_2609-2610) also demonstrated higher expression in R13. Comparatively, the primary alcohol dehydrogenase gene Cbei_1722 was at the same expression level in R13 and R24, while another primary alcohol dehydrogenase gene Cbei_2181 demonstrated two-fold higher expression in R13. More details concerning the expression dynamics of these two primary alcohol dehydrogenase genes in the two reactors will be discussed later. Another alcohol dehydrogenase gene (Cbei_2243) was also expressed much higher in R13 than in R24. It warrants further investigation as to whether this gene plays an important role for solvent production during the ABE fermentation. R13 corresponded to the middle of the exponential phase in R1 when there was active cell growth and metabolism, which demonstrated by the higher expression of several small molecule and cation transport genes (Cbei_0306, _1524, _2122, _3064). On the other hand, R24 corresponded to the late exponential phase in R2, immediately before the cells switched to slow growth and sporulation, and thus it is not surprising that many sporulation related genes (and several stress responsive genes) were much more highly expressed in R24 than in R13 (Additional file 3: Table S1). It is also worth noting that in the case of R2, the sporulation was induced at almost the same time as the onset of solventogenesis; while in R1, since the solvent production was induced from the middle exponential phase, although some early sporulation genes (such as Cbei_0813, _0814, _0097, _1120) were induced after the onset of solvent production, they reached the peak levels several hours later and induced the later sporulation events. In this sense, the induction of solventogenesis and sporulation in R1 appears slightly uncoupled. Interestingly, in R24, there were also several PTS-encoding genes (Cbei_4558-4560, and _2665) and other carbon source metabolism genes (Cbei_4842, _0089, _1789, _3039, _4295) that were more highly expressed than those in R13. These genes may be associated with carbon storage (for example, granulose formation) activities prior to the cells entering the more severe environment during stationary phase.

### Expression of glycolysis genes

**Figure 4:**
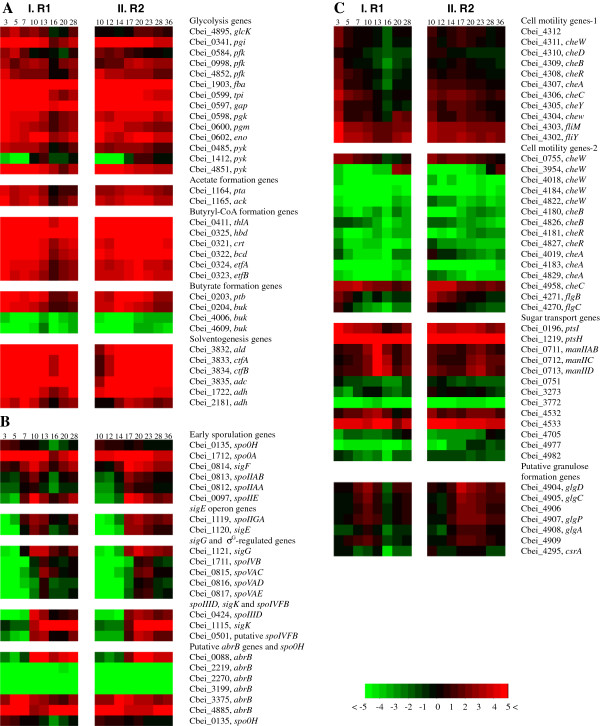
**Comparative time course expression profiles of important genes during the batch fermentation in R1 and R2.** Each corresponding sampling time point was indicated above the heatmap. **(A)** Genes related to glycolysis, acidogenesis and solventogenesis. **(B)** Sporulation genes, putative *abrB* genes and putative sensory kinase genes. **(C)** Genes related to cell motility, sugar transport and granulose formation.

As shown in Figure 4A, expression of the glycolytic genes in both reactors showed very similar patterns, except that the corresponding gene transcription in R2 is about 10 h later than that observed in R1. Similar to what we observed previously [9], expression of most of these genes was down-regulated by 2- to 6-fold in R1 after 16 h and by 2- to 3-fold in R2 after 28 h. However, *glcK* (Cbei_4895) in R2 demonstrated a very different expression pattern, which was highly up-regulated by 2.5-fold after 20 h and maintained at high levels during stationary phase. The reason for this unexpected expression pattern of *glcK* in R2 warrants further investigation. The three 6-phosphofructokinase (pfk) encoding genes (Cbei_0584, Cbei_0998 and Cbei_4852) demonstrated similar expression patterns in both fermentations. While Cbei_0584 was down-regulated after the culture entered late exponential phase (10 h for R1 and 20 h for R2 respectively), the expression of Cbei_0998 and Cbei_4852 maintained at high levels until the initiation of stationary phase. Among the three pyruvate kinase (pyk) encoding genes (Cbei_0485, Cbei_1412 and Cbei_4851), in both reactors, the expression level of Cbei_0485 and Cbei_4851 decreased by 2- to 6-fold after entering stationary phase, whereas Cbei_1412 was expressed at a relatively higher level throughout the late exponential and stationary phases with very low expression levels during early exponential phase. These results suggest that different allelic genes may play very different roles during different growth phases in the fermentation. The expression patterns of the glycolytic genes observed here are generally similar to those described earlier when the transcriptome of the same strain was examined during a batch fermentation using a different growth medium [9].

### Expression of acidogenesis and solventogenesis genes

In the fermentation containing supplemented butyrate (R1), the genes involved in acid formation pathways, including *pta* (Cbei_1164) and *ack* (Cbei_1165) for acetic acid formation, *ptb* (Cbei_0203) and *buk* (Cbei_0204) for butyrate formation, and the genes related to acetoacetyl-CoA and butyryl-CoA synthesis were up-regulated from the very beginning of the fermentation, and declined 3- to 8-fold at 16 h during the late exponential phase corresponding to the time point when the accumulation of acetic acid reached maximum (Figures 4A and 2A). In the unsupplemented control fermentation (R2), the acid formation pathway genes were up-regulated from the middle of exponential phase and maintained at the highest expression levels until the onset of stationary phase (Figure 4A). The addition of butyrate triggered solvent production in R1 as early as the mid exponential phase. Even after the onset of solvent production, acetic acid still accumulated resulting from the active acetic acid synthesis activity. Correspondingly, the expression of the acetic acid formation genes was maintained at high levels until the late exponential phase. Previously, a metabolic flux analysis of the acid formation pathway indicated that the butyrate uptake fluxes in *C. acetobutylicum* were not correlated with acetone production, suggesting that butyrate synthesis enzymes might be also responsible for butyrate re-assimilation [13]. Recently, further experimental evidence was provided that butyrate is not re-assimilated via the adc/ctfAB-dependent pathway as is the case for acetate uptake in *C. acetobutylicum* because the distinct uptake of butyrate has been observed in fermentations with both adc-negative and ctfA-negative mutants [14]. In this study, although butyric acid accumulation was not observed in R1 after initiation of the fermentation, the butyrate synthesis genes were highly up-regulated from the start of the fermentation. This suggests from a different point of view that butyrate formation genes may also be responsible for butyrate re-assimilation in *C. beijerinckii*. In the unsupplemented control fermentation, the expression of the acid formation genes were slightly elevated from the middle of exponential phase until the onset of stationary phase, which also corresponds to the accumulation of acids in R2. In contrast to the fermentation in R1 containing supplemented butyrate, the fermentation in R2 took a longer time to accumulate acids and then switched to solventogenesis.

Based on the RNA-Seq sequencing data, a *sol* operon organized in the order of *ald* (Cbei_3832, encoding aldehyde dehydrogenase)-*ctfA* (Cbei_3833)-*ctfB* (Cbei_3834)-*adc* (Cbei_3835) was revealed in *C. beijerinckii* 8052 [15]. Highly coordinated expression patterns were observed for the *sol* operon genes in both reactors. In R1, corresponding to the early initiation of solvent production in exponential phase, the expression of these genes was high from the very beginning of the fermentation. Expression was further up-regulated by 3- to 5-fold at 7 h and maintained at very high levels throughout the fermentation process, while in R2, the expression was up-regulated by 20- to 50-fold right before the onset of solvent production at 14 h and maintained at highest levels thereafter (Figure 4A).

Two iron-containing alcohol dehydrogenase genes (*adh*, Cbei_1722 and _2181) were inferred to play key roles during primary alcohol production in *C. beijerinckii* 8052 by comparing them with their previously characterized orthologs in *C. beijerinckii* NRRL B592 [9,16,17]. In R1, both genes were induced to high levels from the very beginning, and decreased by 2- to 3-fold before entering the stationary phase. In R2, their expression was up-regulated by 3- to 5-fold at 14 h and kept at highest levels afterwards with a slight decrease after entering stationary phase (Figure 4A). Comparatively, in R1 both *adh* genes (Cbei_1722 and _2181) were transiently up-regulated for less than 10 hours from the beginning of the fermentation, while in R2 the expression of both genes was maintained at very high levels throughout the fermentation after they were induced, corresponding to the continuous solvent accumulation in R2. In addition, Cbei_1722 was comparatively higher regulated than Cbei_2181 in both reactors (2- to 6-fold higher in R1 and 4- to 22-fold higher in R2, respectively). It warrants further investigation whether this implies different significance or functions of these two genes for primary alcohol production in *C. beijerinckii*.

There are 20 annotated alcohol dehydrogenase genes in *C. beijerinckii* 8052, consistent with the great potential this strain has for solvent production [9]. Many of these genes are expressed at high levels corresponding to the solvent production in both reactors. Besides Cbei_1722 and _2181, several other active alcohol dehydrogenase genes in both fermentations include Cbei_0685, _0869, _1932, _2243, _2421, _3864, _3890 and _4354 (Additional file 4: Table S2).

### Expression of sporulation genes

In *B. subtilis*, the gene *spo0H* encodes σ^H^, the earliest acting sigma factor associated with sporulation, which regulates the early sporulation genes, including *spo0A* and *sigF* operon [18]. In this study, the expression of *spo0H* (Cbei_0135) in R1 was induced during early exponential phase, and was down-regulated 2.5-fold at 7 h and further down-regulated later in the fermentation. In R2, a similar expression pattern was observed for *spo0H* where it was down-regulated slightly after 17 h (Figure 4B). The expression of *spo0A* (Cbei_1712) in R1 was induced early during fermentation, and elevated by 2.4-fold at 7 h; afterwards the expression was down-regulated by 5-fold at 13 h. In R2, the transcription of *spo0A* was rather constant at high levels prior to 14 h, and further up-regulated by 3.7-fold at 17 h and maintained at the highest levels until 28 h, after which it was down-regulated by 7.5-fold. It has been reported that in *C. acetobutylicum*, *spo0H* and *spo0A* were both constitutively expressed at constant levels throughout the growth cycle, and the amount of *spo0H* transcript was much lower than that of *spo0A*[19]. This is not consistent with the results for *C. beijerinckii* 8052 obtained in this study, where in both reactors the expression of *spo0H* tended to decline slightly corresponding to the initiation of solvent production, while *spo0A* was up-regulated corresponding to the solvent production and down-regulated before entering the stationary phase. However, in all cases the transcription level of *spo0A* was much higher than that of *spo0H* (Figure 4B).

The *sigF* operon (*spoIIAA*-*spoIIAB*-*sigF*) encodes the anti-anti-sigma factor (SpoIIAA), the anti-sigma factor (SpoIIAB) and the sigma factor σ^F^. In R1, the *sigF* operon genes were up-regulated coordinately by around 4-fold at the onset of solvent production and down-regulated after 16 h. In R2, the expression of the *sigF* operon genes increased by 5- to 9-fold before the onset of solvent production (17 h) and declined by 5- to 6-fold before entering the stationary phase (28 h) (Figure 4B).

σ^E^ is the first mother cell-specific sigma factor in *B. subtilis*[20]. In *C. acetobutylicum*, the expression of *sigE* operon (*spoIIGA*-*sigE*, CAC1694 and CAC1695) does not exceed 1.3-fold higher than that at the initiation of fermentation [21]. In this study with *C. beijerinckii*, the expression of *sigE* operon in R1 (*spoIIGA*-*sigE*, Cbei_1119 and Cbei_1120) was up-regulated around 27-fold at 7 h, and decreased by 3- to 8-fold after 16 h. In R2, the expression of *sigE* operon was up-regulated by 32- and 90-fold for *spoIIGA* and *sigE* respectively at 17 h, and decreased slowly to lower levels after 23 h (Figure 4B). The expression patterns of *sigE* operon in this study further confirmed the fact that forespore and endspore developed more rapidly in *C. beijerinckii* than in *C. acetobutylicum*, as previously discussed [9,17].

In *B. subtilis*, *sigK* gene is expressed from a σ^E^-dependent promoter, and regulated by another transcription factor *spoIIID*[22-24]. The *sigK* gene is expressed as an inactive pro-σ^k^ factor first, and then cleaved to be activated by a membrane-localized protease, SpoIVFB, whose activity is induced by SpoIVB, the signaling protein produced in the forespore [25,26]. In this study, in R1 the expression of *spoIIID* (Cbei_0424) and putative *spoIVFB* (Cbei_0501) was induced at 10 h, and then down-regulated to lower levels after 16 h (7.3- and 6.4-fold lower for *spoIIID* and *spoIVFB*, respectively), while *sigK* (Cbei_1115) was induced at 10 h and further up-regulated by 5.1-fold at 13 h, and maintained at the highest levels throughout the fermentation. In R2, all three genes were induced at the onset of solvent production at 17 h, and further up-regulated at 20 h. Thereafter, the expression of *spoIIID* and putative *spoIVFB* was slightly down-regulated to lower levels while the expression of *sigK* was maintained at the highest levels throughout the fermentation (Figure 4B).

In *B. subtilis*, *abrB* encodes a transition state regulator, whose transcription depression by Spo0A leads to a burst of σ^H^ synthesis at the initiation of sporulation [27]. Maximal expression of *abrB* in *B. subtilis* was observed 2 h before the onset of sporulation [28]. In *C. beijerinckii* 8052, six loci were annotated as genes encoding AbrB family transcriptional regulator. Similar to what we observed previously [9], the expression of three (Cbei_2219, Cbei_2270 and Cbei_3199) is at very low levels most of the time in both R1 and R2. Expression of Cbei_0088 was up-regulated at 10 h and 17 h for R1 and R2 respectively, and maintained at high levels afterwards. The expression of Cbei_3375 and Cbei_4885 was up-regulated before the onset of solvent production, and then decreased to lower levels in both reactors. So, as discussed previously [9], none of the *abrB* genes in *C. beijerinckii* 8052 were observed to display exact antagonistic expression patterns to that of *spo0H*.

Generally, the sporulation genes in both reactors went through similar temporal expression patterns with much faster down-regulation in R1. In addition, after entering stationary phase, most of the sporulation genes demonstrated lower expression levels in R1 than in R2, corresponding to the much faster solventogenesis and sporulation events in R1.

### Expression of cell motility genes

The cell motility-related genes encode products associated with chemotactic responses and flagellar assembly [29]. It was confirmed that in *C. beijerinckii* 8052 a flagellar/chemotaxis multi-gene cluster (Cbei_4312-4302) exists similar to that in *C. acetobutylicum* (CAC2225-2215) [15,30]. In R1, most genes in the flagellar/chemotaxis cluster were down-regulated by 2- to 4-fold at 5 h and then decreased further to even lower levels. In R2, although low expression levels were observed as early as 12 h for Cbei_4312, _4311, _4309 and _4308, expression of the genes in the flagellar/chemotaxis cluster declined after 20 h when noticeable solvents were detected. Comparatively, *fliM* (Cbei_4303) and *fliY* (Cbei_4302) had much higher expression levels than the others in the cluster, although they were also down-regulated coordinately within the same time frame (Figure 4C).

There are several other genes in *C. beijerinckii* 8052 annotated as motility-related, although most of them are not actively expressed during the fermentation process (Figure 4C). Among them, Cbei_0755 (*cheW*) in both reactors gradually declined to lower levels after the onset of solvent production. Another *cheW* gene (Cbei_3954) exhibited an unexpected antagonistic pattern, which was highly expressed after the cell culture entered stationary phase and reached maximum finally in both reactors. This observation is identical to that reported previously [9]. In both reactors, expression of gene Cbei_4958 (*cheC*) was maintained at high levels throughout the fermentation, although it was down-regulated slightly after solvents were accumulated. The flagellar motility-related genes *flgB* (Cbei_4271) and *flgC* (Cbei_4270) were strongly expressed during early growth phases (before 7 h and 17 h in R1 and R2, respectively), and down-regulated to very low levels later in the fermentation (Figure 4C).

### Expression of putative granulose formation genes

Granulose is a glycogen-like polymer used by bacteria for energy storage under severe environments [31]. In *C. beijerinckii* 8052, the RNA-Seq data indicated that *glgC* (Cbei_4905, encoding glucose-1-phosphate adenylyltransferase) and *glgD* (Cbei_4904, encoding ADP-glucose pyrophosphorylase) were organized in an operon, while the gene encoding 1,4-alpha-glucan branching enzyme (Cbei_4909), *glgA* (Cbei_4908, encoding glycogen synthase), *glgP* (Cbei_4907, encoding a granulose phosphorylase and functioning to depolymerize granulose) and the gene encoding the catalytic region of alpha amylase (Cbei_4906) were organized in another adjacent operon [15]. The putative granulose formation genes (Cbei_4904-4909) in *C. beijerinckii* 8052 were highly coordinately expressed in both reactors; they were up-regulated at the onset of solvent production, and maintained at highest levels until the transition from exponential phase to stationary phase, corresponding to peak levels of the expression for PTS transporter genes as discussed above. The expression level of *csrA* (Cbei_4295, encoding carbon storage regulator) was much lower than that of the other granulose formation genes (Figure 4C).

## Discussion

With 40 mM sodium butyrate supplemented into the ABE fermentation with *Clostridium beijerinckii* 8052, solvent production was triggered about 10 h earlier, butanol titer, yield and productivity were all significantly improved. These results demonstrated significant potential application values for commercial butanol production. Butyric acid and other short chain fatty acids (such as acetic acid) are usually contained in various waste streams from many agricultural and industrial processes. They are often considered as pollutants, and usually detrimental to the fermentation microbes in respective bioprocesses. However, these waste streams can be used as valuable carbon sources to supplement the ABE fermentation media in the commercial fermentation process to enhance the butanol production [32].

Butyrate supplementation in R1 triggered solvent production at mid-exponential phase; even after the initiation of solventogenesis, acetic acid accumulation was still observed in R1 until the late exponential growth phase. This observation does not conform to the classical phenomenological model that acids accumulation usually occurs during the early acidogenesis phase prior to solvent production. Butyrate supplementation apparently caused feedback inhibition to the butyrate production pathway, and thus a decreased flux towards butyrate formation. Since the butyrate formation pathway is also responsible for energy (ATP) generation for the cell culture, the culture needs to find an alternate ATP-generation pathway to make up the energy deficit. One option is the enhancement of the acetate formation pathway resulting in an increase in acetate accumulation in R1 [33]. In addition, the supplemented butyrate also provided a larger carbon feedstock pool for conversion to a higher final total solvent concentration, and resulted in a metabolism pathway shift towards butanol production and a higher butanol/acetone ratio. It is worthwhile to point out that the sugar-based butanol yield in R1 (0.41 g g^-1^ glucose) is near 100% of the maximum theoretical yield of butanol production from glucose [34], indicating that the supplemented butyrate was re-assimilated for butanol production. In R1, 2.4 g/L (or 32.4 mM) more butanol has been produced compared to that in R2. Although the addition of butyrate has enhanced butanol production, the supplemented 40 mM butyrate has not led to an equivalent 40 mM butanol production improvement on a carbon-per-carbon basis.

With butyrate addition in R1, the expression of the genes in central metabolism related to solventogenesis events were induced earlier and accelerated. In addition, the culture in R1 showed elevated maximum expression levels for butyryl-CoA and solvent formation genes (although they corresponded to very different time frame than in R2) in comparison to that in R2, which may correlate with the faster and higher solvent production. In R1, the sporulation events were uncoupled from the induction of solventogenesis. In both reactors, the sporulation genes went through a similar temporal expression patterns to those observed in *B. subtilis* and *C. acetobutylicum*, although usually down-regulated faster in R1 than the control in R2, corresponding to a faster and stronger sporulation. The motility related genes were generally down-regulated to lower levels before the culture entered the stationary phase in both reactors. However, in R2 this process took much longer and the gene expression was maintained at comparatively higher levels after entering stationary phase. This observation was consistent with the continuous solvent accumulation during stationary phase and lower final solvent titer in R2. Spo0A has been reported to negatively regulate the expression of chemotaxis/motility genes [19,35,36]. In R1, stronger induction of *spo0A* was observed with the onset of solvent production, which may coincide with the more rapid down-regulation and lower final levels of expression of the motility-related genes.

**Figure 5:**
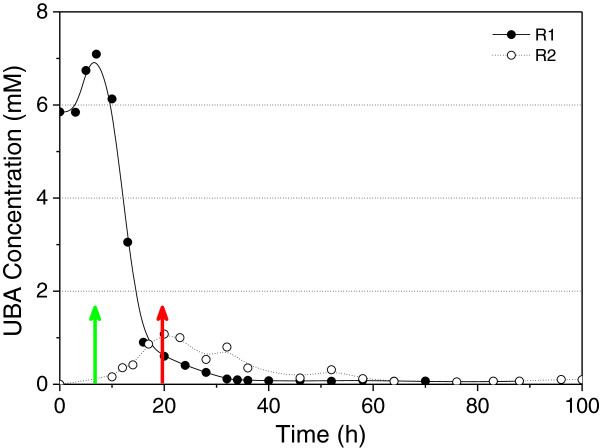
**Calculated undissociated butyric acid (UBA) levels over time in R1 and R2 based on the measured butyrate concentration.** The onset of solvent production was indicated by green arrow (for R1) and red arrow (for R2), respectively.

The concentration of undissociated butyric acid (UBA) has been shown as a controlling factor involved in the switch from acid to solvent production in the solventogenic clostridia [2]. Monot et al. reported that, when the concentration of UBA reached a level of 0.5 to 1.5 g L^-1^, solvent production was initiated [37]. Husemann and Papoutsakis determined that 6–13 mM of UBA was required for the initiation of solventogenesis when the external pH was between 3.7 and 5.0 [4]. However, Harris et al. later suggested that butyrate may not be involved in the induction of solventogenesis in a study where they obtained high solvent production from the fermentation with a butyrate kinase inactivated mutant of *C. acetobutylicum*[33]. Zhao et al. further indicated that butyryl phosphate (but not acetyl phosphate) correlated with (and possibly regulated) the initiation of solvent formation through a sensitive measurement of butyryl phosphate and acetyl phosphate levels in the wild type *C. acetobutylicum* ATCC 824 and several mutant strains; transcriptional analysis further demonstrated that high butyryl phosphate levels also corresponded to down-regulation of chemotaxis and motility genes and up-regulation of solvent formation genes [38]. In this study, since the pH of the fermentation was known at each sampling point, the UBA level could be calculated using the Henderson–Hasselbalch equation [4,33]. Based on the assumption that undissociated acids can freely cross the cell membrane, the intracellular and extracellular undissociated acid concentrations should be identical [33]. As shown in Figure 5, the peaks of UBA levels in both reactors corresponded well to the onset of solvent production. However, the peak level in the control (1 mM) was about six-times lower than that in the fermentation with butyrate supplementation (7 mM), and thus it is unlikely that there is a specific threshold level of UBA for the induction of solventogenesis in *C. beijerinckii*, although the latter falls well into the range of 6–13 mM as suggested by the established phenomenological models for initiation of solvent production in *C. acetobutylicum*[3,4]. Instead, butyryl phosphate may be associated with the initiation of solvent production as suggested by Zhao et al. [38]. With the exogenous butyrate supplementation in R1, the high level of intracellular butyrate may result in an elevated concentration of butyryl phosphate either through feedback inhibition to the butyrate formation pathway or through re-assimilation of butyrate converting to butyryl phosphate through the reversed butyrate formation pathway [14]. While in R2, ATP was generated through the acid (both acetic and butyric) formation pathway during acidogenesis phase, thereby leading to a much higher ATP level (or ATP/ADP ratio) when compared to that in R1. Therefore, although the level of UBA was much lower in R2, through the equilibrium of the reaction for the conversion between butyrate and butyryl phosphate, at the onset of solvent production the intracellular butyryl phosphate level (the “threshold” level) in R2 may actually approximate that in R1. This also is in accord with the observation that the solventogenic switch occurred at a much higher cell density level in R2 than that in R1, because the culture in R2 needed to accumulate more ATP (or a higher ATP/ADP ratio) before switching to solventogenesis, and thus meanwhile produced more cell biomass. Spo0A has been suggested as the master regulator for solventogenesis and sporulation events in clostridia and bacilli, and the phosphorylated Spo0A has been found to regulate the expression of various target genes [19,35,36]. The elevated level of butyryl phosphate beyond a “threshold level” may donate its phosphate group to Spo0A, and thereby trigger solventogenesis. In addition, the phosphorylated Spo0A may also induce sporulation and a reduction of cell motility.

Actually, butyrate kinase from *C. acetobutylicum* has been reported to exhibit reversible activities, and the discrepancies in specific activities in the two directions suggest that there may be multiple forms of the enzyme [2,39,40]. In *C. beijerinckii*, there are three genes (Cbei_0204, _4006 and _4609) annotated to encode butyrate kinase. Besides Cbei_0204 which is co-operonic with *ptb* and highly regulated during the fermentation, Cbei_4006 and _4609 had comparatively only negligible expression levels throughout the fermentation process in both reactors (Figure 4A). It still warrants further study in *C. beijerinckii* as to whether butyrate is re-assimilated through the reversible butyrate formation pathway and whether Cbei_0204 is responsible for this reversible reaction. In addition, further study is also required to elucidate the detailed molecular mechanism for solvent production in the ABE fermentation. Development of advanced analytical methods for precise measurement of the intracellular fermentation intermediates, and generation of mutant strains with altered product profiles through metabolic engineering would be helpful to address this problem.

## Conclusions

With the butyrate supplementation for the ABE fermentation with *Clostridium beijerinckii* 8052, the solvent production was triggered early in the mid-exponential phase and completed more rapidly than the unsupplemented control. Butyrate addition also led to a significant improvement in the final butanol titer, sugar-based yield and productivity. The butanol/acetone ratio increased to 2.4 versus 1.8 in the control, indicating a metabolic shift towards butanol production. Genome-wide transcriptional analysis employing RNA-Seq technology indicated that, compared to the control, in the fermentation with butyrate supplementation, the gene expression related to solventogenesis was induced about 10 hours earlier, and the up-regulation of sporulation genes was delayed and uncoupled from the solventogenesis events. The undissociated butyric acid profile from the fermentation indicated that the supplemented butyrate provided feedback inhibition to butyrate formation and may be re-assimilated through the reversed butyrate formation pathway, thus resulting in an elevated level of intracellular butyryl phosphate; this may have acted as a phosphate donor to Spo0A and subsequently triggered the solventogenesis and sporulation events.

## Materials and methods

### Bacterial culture and fermentation experiment

Laboratory stocks of *C. beijerinckii* 8052 spores were stored in sterile H_2_O at 4°C [41]. Spores were heat-shocked at 80°C for 10 min, followed by cooling on ice for 5 min. The heat-shocked spores were inoculated at a 1% inoculum level into tryptone–glucose–yeast extract (TGY) medium containing 30 g L^-1^ tryptone, 20 g L^-1^ glucose, 10 g L^-1^ yeast extract and 1 g L^-1^ L-cysteine. The TGY culture was incubated at 35 ± 1°C for 12–14 h in an anaerobic chamber under N_2_:CO_2_:H_2_ (volume ratio of 85:10:5) atmosphere. Subsequently, actively growing culture was inoculated with a 5% ratio into two BioFlo® 310 benchtop bioreactors (New Brunswick Scientific Co., Enfield, CT) both containing sterilized solution with 60 g L^-1^ glucose, 1 g L^-1^ yeast extract and chemically modified P2 medium (MP2) [42]. In the first reactor (R1), 40 mM (3.52 g L^-1^) sodium butyrate was also included in the medium to test the effect of butyrate on solvent production comparing to the control in the second reactor (R2). MP2 medium, which was modified from P2 medium [43], contained following compounds (in g L^-1^): KH_2_PO_4_, 0.5; K_2_HPO_4_, 0.5; (NH_4_)_2_SO_4_, 2; MgSO_4_ · 7H_2_O, 0.2; MnSO_4_ · H_2_O, 0.01; FeSO_4_ · 7H_2_O, 0.01; NaCl, 0.01; *p*-Aminobenzoic Acid, 0.001; Thiamin-HCl, 0.001; Biotin, 0.00001. 2-(N-Morpholino)ethanesulfonic acid (MES) (100 mM) (Sigma-Aldrich Co. LLC, St. Louis, MO) was added to the fermentation medium to prevent overacidification [42]. Oxygen-free nitrogen was flushed through the broth to initiate anaerobiosis until the culture initiated its own gas production (CO_2_ and H_2_). Temperature was controlled at 35 ± 1°C. A stirring at 50 rpm was employed for mixing. The pH value was monitored and recorded throughout the fermentation process using the BioCommand® supervisory software (New Brunswick Scientific Co., Enfield, CT) equipped with the reactors. Cell density and product concentration were monitored through the course of fermentation. For sequencing purpose, samples were taken over the exponential and stationary phases from both R1 and R2 (Figure 1 and Table 1).

### Culture growth and fermentation products analysis

Culture growth was measured by following optical density (OD) in the fermentation broth at A_600_ using a Varian’s Cary 300 Bio UV-visible spectrophotometer (Agilent Technologies, Inc., Santa Clara, CA). ABE, acetic acid, and butyric acid concentrations were quantified using gas chromatography (GC) system as previously described [15]. Butanol yield (g g^-1^) was defined as total butanol produced divided by the total sugar utilized, although the supplemented butyrate was also used as the substrate in R1; butanol productivity was calculated as total butanol produced divided by the fermentation time and is expressed in g L^-1^ h^-1^.

### RNA isolation, library construction and sequencing

In preparation for RNA isolation, 10 ml cultures were harvested at each time point, and centrifuged at 4,000 × g for 10 min at 4°C. Total RNA was extracted from the cell pellet using Trizol reagent based on manufacturer’s protocol (Invitrogen, Carlsbad, CA) and further purified using RNeasy minikit (Qiagen, Valencia, CA). DNA was removed using a DNA-*free*™ kit (Ambion Inc., Austin, TX). RNA quality was assessed using a nanochip on a model 2100 bioanalyzer (Agilent Technologies, Santa Clara, CA). RNA concentration was determined with a nanodrop (Biotek Instruments, Winooski, VT). Bacterial 16S and 23S ribosomal RNAs were removed with Ribo-Zero™ rRNA Removal Kits from Epicentre (Madison, WI) following the manufacturer’s protocol. The enriched mRNA was converted to a RNA-Seq library using the mRNA-Seq library construction kit (Illumina Inc., San Diego, CA) following manufacturer’s protocols. For sequencing, six samples were pooled and sequenced on one single lane of an eight-lane flow cell with the HiSeq 2000 system (Illumina Inc., San Diego, CA). The derived sequence reads were 100 nt long. The overall error rate of the control DNA was 0.84%. The total number of reads generated from each library is summarized in Table 1.

### RNA-Seq data analysis

The generated 100-nt reads were mapped to the *C. beijerinckii* 8052 genome using MAQ, and those that did not align uniquely to the genome were discarded [44]. The quality parameter (−q) used in MAQ pileup was set to 30, which implies there is a 1 in 1000 probability that the read is incorrectly mapped [44]. Each base was assigned a value based on the number of mapped sequence coverage. The quantitative gene expression value, RPKM (reads/Kb/Million), was calculated using custom Perl scripts by normalizing the sequence coverage over the gene length and total unambiguously mapped reads in each library [8,45].

The gene expression results were visualized colorimetrically using heatmap plots. R language and the open source software Bioconductor based on R were used for the data analysis and plots [46-48]. Time course RPKM values were first transformed to log_2_-scale. The log_2_-transfromed RPKM values were then properly centered for better representation of the data by the heatmap plots. More details about this method were summarized in Additional file 5 in the supplemental material.

### RNA-Seq sequencing data

The RNA-Seq sequencing data have been deposited in the NCBI Sequence Read Archive (SRA) under the accession number SRA101395.

## Competing interests

The authors declare that they have no competing interests.

## Authors’ contributions

YW, XL, HPB conceived and designed the study. YW and XL performed the experiments. YW and XL analyzed the RNA-Seq data. YW, XL and HPB wrote the manuscript. All authors read and approved the final manuscript.

## Supplementary Material

Additional file 1: Figure S1 Correlation of gene expression in sample R23 (took at 14 h from R2) with its replicate sample R23-2 (took at 14 h from a fermentation operated under identical conditions as for R2). Click here for file

Additional file 2: Figure S2 Differentially expressed genes at the induction of solventogenesis in R1 (7 h) and R2 (17 h) respectively (The labeled numbers on the figure are the gene ID numbers; all the genes in the figure are listed in Additional file 3: Table S1; refer to Table S1 for more details). The points above the “Y = X + 2” line represented the differentially more highly expressed genes in R24, while the points below the “Y = X-2” line represented the differentially more highly expressed genes in R13. Click here for file

Additional file 3: Table S1 Differentially expressed genes at the induction of solventogenesis in R1 (7 h) and R2 (17 h) respectively. Click here for file

Additional file 4: Table S2 The expression of the putative alcohol dehydrogenase genes in *C. beijerinckii* 8052. Click here for file

Additional file 5 RNA-Seq data analysis. Click here for file
