# Supplementary material for: Effects of supplementary butyrate on butanol production and the metabolic switch in Clostridium beijerinckii NCIMB 8052: genome-wide transcriptional analysis with RNA-Seq
Source: Biotechnol Biofuels. 2013 Sep 27;6:138. doi: 10.1186/1754-6834-6-138 (PMC3849199; doi:10.1186/1754-6834-6-138)
Supplement: Additional file 1: Figure S1 — Correlation of gene expression in sample R23 (took at 14 h from R2) with its replicate sample R23-2 (took at 14 h from a fermentation operated under identical conditions as for R2). [file 1754-6834-6-138-S1.doc]

**Figure S1 Correlation of gene expression in sample R23 (took at 14 h from R2) with its replicate sample R23-2 (took at 14 h from a fermentation operated under identical conditions as for R2).**
