# Supplementary material for: Effects of supplementary butyrate on butanol production and the metabolic switch in Clostridium beijerinckii NCIMB 8052: genome-wide transcriptional analysis with RNA-Seq
Source: Biotechnol Biofuels. 2013 Sep 27;6:138. doi: 10.1186/1754-6834-6-138 (PMC3849199; doi:10.1186/1754-6834-6-138)
Supplement: Additional file 2: Figure S2 — Differentially expressed genes at the induction of solventogenesis in R1 (7 h) and R2 (17 h) respectively (The labeled numbers on the figure are the gene ID numbers; all the genes in the figure are listed in Additional file 3: Table S1; refer to Table S1 for more details). The points above the “Y = X + 2” line represented the differentially more highly expressed genes in R24, while the points below the “Y = X-2” line represented the differentially more highly expressed genes in R13. [file 1754-6834-6-138-S2.doc]

**Figure S2 Differentially expressed genes at the induction of solventogenesis in R1 (7 h) and R2 (17 h) respectively (The labeled numbers on the figure are the gene ID numbers; all the genes in the figure are listed in Table S1; refer to Table S1 for more details). The points above the “Y = X+2” line represented the differentially more highly expressed genes in R24, while the points below the “Y = X-2” line represented the differentially more highly expressed genes in R13.**
