# Supplementary material for: Effects of supplementary butyrate on butanol production and the metabolic switch in Clostridium beijerinckii NCIMB 8052: genome-wide transcriptional analysis with RNA-Seq
Source: Biotechnol Biofuels. 2013 Sep 27;6:138. doi: 10.1186/1754-6834-6-138 (PMC3849199; doi:10.1186/1754-6834-6-138)
Supplement: Additional file 5 — RNA-Seq data analysis. [file 1754-6834-6-138-S5.doc]

## RNA-Seq data analysis

## Time course RPKM values were first transformed to log_2_-scale. The distribution histogram of the data was shown in Figure S1. The data covered a large range and were normally distributed with center at around 5. For better representation of the data by the heatmap plots, the log_2_-transfromed RPKM values were then centered by subtracting 5. The histogram for the distribution of the “5-centered” data was shown in Figure S2. With a color scale ranging from -5 to 5, the heatmap plots represented most of the transformed gene expression data.

**
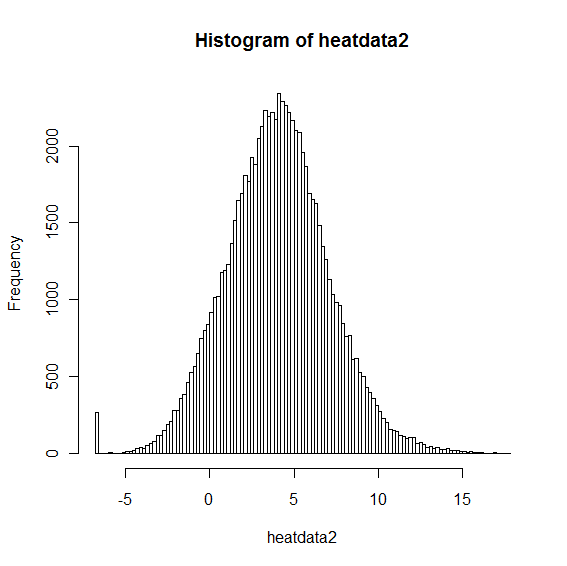
**

**Log_2_ RPKM**

**Figure S3 Distribution of the log_2_-tranformed RPKM values for all the genes**

**
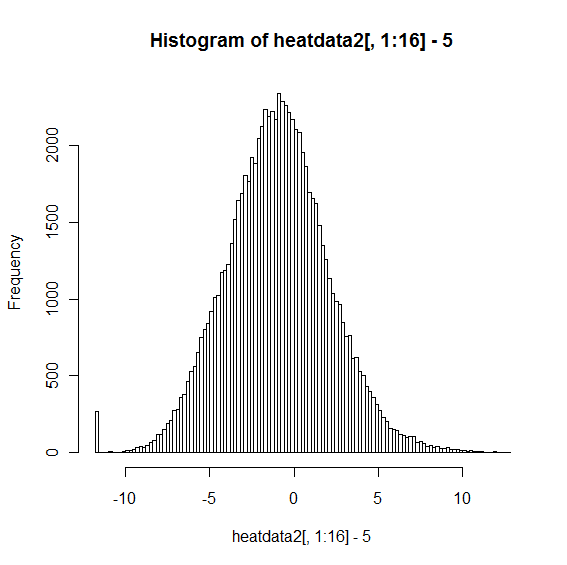
**

**Log_2_ RPKM**

**Figure S4 Distribution of the “5-centered” log_2_-tranformed RPKM values for all the genes**
